# Kurdish Refugee Beliefs about Mental Health and Help-Seeking: A Community-Engaged Research Study in Tennessee

**DOI:** 10.3390/ijerph20021224

**Published:** 2023-01-10

**Authors:** Leah S. Branam, Ismail Yigit, Sipal Haji, Jennifer Clark, Jessica M. Perkins

**Affiliations:** 1 Department of Human and Organizational Development, Peabody College of Education and Human Development, Vanderbilt University, 230 Appleton Pl, Nashville, TN 37203, USA; 2 School of Nursing, Vanderbilt University, 461 21st Ave S, Nashville, TN 37240, USA; 3 Department of Sociology, Tennessee State University, 3500 John A Merritt Blvd, Nashville, TN 37209, USA; 4 Catholic Charities, Diocese of Nashville, 2806 McGavock Pk, Nashville, TN 37214, USA

**Keywords:** refugees, mental health, community-engaged research

## Abstract

Refugee populations exhibit high rates of PTSD, anxiety, depression, and psychological distress, but are less likely to receive care than the general population. Perceptions among the Kurdish refugee community about causes and consequences of mental illness symptoms and perceived barriers to help-seeking are understudied. This community-engaged research study conducted in-depth interviews with Kurdish refugees from Iraq to explore their beliefs about drivers of mental illness and seeking help for mental health. Iterative thematic analysis of transcripts from ten participants indicated four key themes: (1) social network loss due to resettlement causes poor mental health; (2) socioeconomic status loss due to unrecognized professional qualifications puts strain on mental health; (3) social stigma about mental health and fears about disclosure of mental health issues within community and subsequent negative gossip prevent help-seeking; and (4) social interaction may alleviate mental illness symptoms. Overall, Kurdish refugees perceived social factors as major drivers of mental illness symptoms and barriers to help-seeking in their community. However, while participants believed that the general community attitude was against help-seeking, most participants personally expressed support of anyone in their community needing to see a mental health professional. Future research should assess the extent to which perceived community norms differ from aggregated personal help-seeking attitudes and behaviors among Kurdish refugees from Iraq in the United States.

## 1. Introduction

By the end of 2021, significant hardships such as war, conflict, targeted persecutions, and natural disasters had displaced more than 89.3 million persons across the world [1]. At least 53.2 million individuals had been internally displaced within their countries of origin, while 21.3 million refugees had been externally displaced within new countries [1]. On average, refugees exhibit symptoms of post-traumatic stress disorder (PTSD) at much higher rates than in general populations [2]. Exposure to traumatic events leading to displacement or during displacement can cause psychological stress, depression, and PTSD [3,4]. To provide culturally responsive care, programs serving refugees need to understand clients’ perceptions about mental health causes, consequences, and needs, which may vary across refugee sub-populations.

The United States resettled about 60,000 refugees in 2021 [5] from a range of national, cultural, socioeconomic, professional, historical, and trauma backgrounds. Since the Gulf War and Iraq War, the United States has resettled a mixed group of about 103,000 refugees from Iraq [6]. Past research has found that almost one-third of Iraqi refugees are at risk for PTSD and approximately 50% exhibit anxiety, depression, and emotional distress [7]. Mental health stigma among Muslim immigrant populations within the US may prevent access to support [8]. However, few studies have focused on Kurdish refugees from Iraq who fled from targeted persecution at four different time points since the Gulf War [9].

### 1.1. Kurdish Refugees and Refugee Mental Health Challenges

Kurdish refugees from Iraq differ in cultural background, language, and set of traumatic experiences compared to other refugee populations from Iraq. The first wave of refugees started with the Kurdish rebellion against Saddam Hussein in 1976. The second wave occurred after Islamic Republic of Iran was established in 1979. The third wave fled Iraq in response to genocidal attacks by Saddam Hussein against all Kurds in Iraq in the late 1980s. The last wave of refugees fled when harsh policies targeted Kurdish intellectuals in 1996 [9]. Thus, the Kurdish refugee population in the United States includes many highly educated persons with professionally successful backgrounds, has experienced multiple persecutions and displacements, and represents a minority religion experiencing discrimination.

This background may differently influence Kurdish refugee perceptions about mental health symptoms, associated challenges, and needed supports, as compared to other major refugee communities within the United States. Culturally sensitive treatment may also not be available because many providers are not familiar with or educated in the specific health needs of different refugee groups [10]. Moreover, mental health services often approach the concept of mental health from a Westernized perspective. Assessment and treatment focus on biological and psychological aspects of mental health rather than the social and structural determinants of health [10]. Few studies among the Kurdish diaspora in the United States have documented their perceptions related to mental health [9,11]. Within a recent systematic review of refugee perspectives about mental health and perceived barriers to help-seeking, only two studies included Kurdish representation [12]. Both studies were conducted in the UK, targeted refugee participants from several countries and backgrounds, and collapsed all refugee responses together. However, prior research suggests that perspectives on mental health [13] and about barriers to help-seeking [14] may vary between sub-populations of refugee and immigrant groups.

Most refugees do not receive a mental health specific assessment nor any follow-up mental health support during or after the resettlement process in the United States [12,15,16]. Mental health conditions among refugees remain untreated or under-treated due to many structural and socioeconomic barriers that inhibit refugee access to mental health care [4,17]. These barriers include complex health care information, lack of interpreters, high costs of care, no insurance, low health literacy, and insecure housing [12,18,19]. Cultural influences that do not support seeking care, worries about immigration status, and perceived stigma about seeking mental health treatment also prevent evaluation and care [12].

No studies have specifically assessed US-based Kurdish refugees’ interpretations of mental health issues, nor their perceptions about barriers to help-seeking and potential sources of support. Inviting Kurdish refugees from Iraq to describe their perceptions about mental health beliefs through a community-engaged research process would address a gap in the literature and provide an opportunity to grow research capacity within a refugee community and community organization. The principles of community-engaged research emphasize that health outcomes are rooted in structural and social factors, and engaging community members with lived experience in the research process is the most appropriate approach to understanding and addressing health inequities [20]. This process includes community stakeholder engagement in the development, implementation, analysis and/or dissemination of research.

### 1.2. This Community-Engaged Research Study

An academic research team and a refugee-serving community organization in Nashville, TN, formed a community-engaged research partnership to address the paucity of refugee voices in mental health research. The community organization is a nonprofit social service agency that provides refugee resettlement and case management services. Sequential conversations with the community organization helped the partnership to identify a focus on Kurdish refugee mental health as an area for collaboration. Middle Tennessee hosts a substantial refugee population, with Nashville hosting the largest Kurdish population in the US [21,22]. Currently, more than 15,000 Kurds live in Nashville [23], with over 1500 refugees from Iraq resettling in Tennessee since 2014 [24]. A study of older Kurdish refugees in Nashville found that approximately 67% had symptoms of depression and approximately 26% had symptoms of severe depression [25].

Individuals’ attitudes and behaviors are embedded in and influenced by interacting systems within the ecological environment, including family, faith organizations, community networks, local infrastructure, national policy, societal norms, and structural forces [26]. Moreover, societal structures, such as policy, access to resources, and community settings can influence health outcomes for individuals as social determinants of health [27]. While community partners generally felt equipped to discuss economic and structural causes of mental health burden and barriers to care among refugee communities, conversations among partnership members found that the community partner (and the research literature) lacked a tailored understanding about social factors associated with mental health among the Kurdish community.

Thus, further conversations with members of the Kurdish refugee community, served by the community partner organization, drove the formation of two research questions, specifically, (1) ‘How do members of the Kurdish refugee community describe mental health and mental illness?’, and (2) ‘What are the social factors that facilitate or prevent Kurdish refugees from seeking help for mental health?’. The aims were to increase understanding about Kurdish refugee beliefs about mental health and identify spaces for refugee service programs to design mental wellness support services in culturally responsive ways.

## 2. Materials and Methods

### 2.1. Study Design

The partnership suggested using a qualitative study design to collect data that relied on Kurdish research assistants (RAs) from the local established community to conduct one-on-one in-depth interviews. Community partners emphasized that this format would create a trusting environment in which to gather information, and research partners identified that collecting refugee voices would address a gap in the literature. Additionally, training these research assistants and the community organization in research methods would contribute to the collaborative process and increase their capacity for future projects. The research partners practiced reflexivity [28] throughout the research process by engaging in ongoing discussion about how their social identities may affect study design, implementation, and interpretation. To support study design and plan the research project, the community–academic partnership used a Give-Get Grid tool. This tool helps to establish clarity and expectations around what each partner would bring and receive from the partnership (Table 1) [29]. Prior to recruiting any participants and conducting study procedures, the partnership received approval from the academic partner’s institutional review board to conduct this research.

### 2.2. Participants

Eligibility criteria for study participation included male and female Kurdish household heads who had initially resettled in Nashville within less than five years. If a household had multiple household heads, both were invited to participate in the study. Exclusion criteria included Kurdish individuals under 18 years old or those who had resettled in the US longer than five years ago. Eligible individuals were recruited via a convenience sampling method from the community partner’s known network of Kurdish refugees. The community partner assisted with recruitment by telling eligible participants about the study. Men and women were equally targeted for participation. Twelve potential participants were initially contacted and ten agreed to participate. The final sample included 5 men and 5 women, ages 23–58, and 8 married and 2 single individuals.

### 2.3. Study Procedures

The Principal Investigator trained two members of the Kurdish community in qualitative research methods. Specifically, they were taught how to facilitate interviews and analyze qualitative data, as well as provide feedback on the research process in conjunction with the community partner. The semi-structured interview guide was then designed in consultation with the refugee-serving community organizations, the RAs, and other Kurdish community members (non-participants). The interview guide and informed consent document were translated into Kurdish by one of the RAs and went through multiple rounds of translation—back-translation and piloting.

To recruit participants, a staff member at the community partner organization first contacted eligible adults from their client base, starting with the most recently resettled, to notify them of the study and to ask if they were willing to have a research team member contact them. Then, a RA called an eligible adult on the phone, invited that person to participate in the study, and went through the consent process verbally. If the person agreed to participate, a written consent form was emailed or delivered to their home (per COVID-19 social distancing procedures). The RA also ensured that the interviewee had a private space to speak and set up a time to conduct the one-on-one semi-structured interview over the phone, which typically lasted about an hour. The interview questions focused on the causes and ways to alleviate symptoms of stress, anxiety, and depression, and on attitudes towards help-seeking for mental health with different professionals. The interview guide allowed participants to describe their lived experience of being a member of the Kurdish community and how that context related to mental illness and help-seeking (Appendix A).

Interviews were conducted in Kurdish, audio-recorded, and took place between April and June 2020. Interviewees were offered a 25 USD gift card as compensation for their time. The RAs transcribed and translated all interviews into English so that the study team could collaboratively engage in the analytical process.

### 2.4. Analysis

The study team used an inductive, iterative approach and flexible coding [30] in MAXQDA to conduct thematic analysis of the interview transcripts and generate themes. This analytic process was conducted as a group with the PI, one of the Kurdish RAs, and two additional researchers trained in qualitative research methods. Each individual conducted thematic analyses independently on the transcripts. The full analysis group then met to compare individual themes and come to consensus on final themes. The study team took a pragmatic approach to the interview data by focusing on findings that can be used to design interventions to improve mental health outcomes for the Kurdish refugee population. Findings were shared with members of the study team who are Kurdish and with the community partner and they affirmed the results.

## 3. Results

Ten Kurdish refugees participated in this study (five men and five women). Thematic analysis of participants’ discussions highlighted interpersonal factors as causes of mental health symptoms, barriers to seeking support, and solutions to addressing mental health symptoms. Four key themes arose: social network loss, loss of professional qualifications, fears about community gossip, and social connectedness to improve mental health (Table 2). These themes are discussed below and presented in Table 2.

### 3.1. Social Network Loss Due to Resettlement Causes Poor Mental Health

Many respondents indicated that being far from family or other social ties because of resettlement causes symptoms of stress, anxiety, and depression. One participant shared, “I am one of those people who left their parents. I lost a lot of my friends. The main reasons [for symptoms of mental illness] are those.” According to another participant, “When you first come to a different country being far away from your parents, family, and friends will cause a big impact on you.” Participants reiterated that the loss of social connections through the resettlement process contributes to mental illness.

### 3.2. Loss of Professional Qualifications and Social Status in New Context Puts Strain on Mental Health

Several participants emphasized that loss of professional status can contribute to mental illness. They repeatedly mentioned the loss of professional qualifications as a particular strain on mental health. “You spent twenty years getting your college degree and start all over here. Whatever you had in your own country, you will lose it here, for example, your education and your job,” said one interviewee. Other participants described similar circumstances. They commented that educational qualifications and professional experiences mostly did not translate to the US context. “Back in Kurdistan you have your degree and language, and you know the lifestyle there…Coming here to everything being different and learning a new language is very hard in the beginning. The environment changes, their degree is worth nothing.” Therefore, individuals had to take employment for which they were overqualified and start from scratch in pursuing degrees in their fields of expertise.

### 3.3. Fears about Disclosure to Community and Subsequent Negative Gossip Prevent Help-Seeking

While participants discussed many elements of the resettlement process as factors that can cause stress, anxiety, and/or depression, many also reported several community-level attitudes against help-seeking from a mental health professional. Examples of stigma included perceived negative attitudes toward persons with mental health challenges from the broader community. “Not going to see counselor, maybe I have done some things wrong, and I do not wish people know about that. Or he might think, people will talk behind his back.” Another participant explained that,

“*We have a Kurdish saying that says it’s okay to walk on an empty stomach but dress sharp so it’s not important about how you feel it’s important about how other people look at you. So, this is just a simple example about the Eastern society about how they feel and react. So, when they come here, they don’t want to talk about their issues, and they bring their habits here. So, you will try hard to keep your problems away from people if they find out they might mock and use it against you as a weak point. Therefore, our people they don’t talk about their mental issues*.”

Participants also reported the perception that community members believed mental health treatment to be unhelpful. They suggested that community-level stigma against individuals who experience mental illness prevents people in their community from seeking help from mental health professionals. One participant said, “Maybe that person doesn’t want to visit a mental health professional, so people won’t think that he is mentally ill.” Other participants described similar perceptions of stigma that would influence a person’s help-seeking behaviors.

The perception of stigma was articulated as different within the Kurdish community compared to Western culture.

“*The way our society looks at mental issues is way different than Western communities like Europe or the US. It is so crucial to address this with a specialist mental health doctor. You will be confident about him not sharing your thoughts and behaviors with others. He will keep your secret and won’t make fun of you. [With the exception of doctors], even your closest friend will talk behind your back and will blame you for these [symptoms]. Therefore, mentally ill people will not share and will keep it private. [He is afraid that] people in society will blame him and say he is a psychopath and stay far from him. But in European society, [it] is normal to talk and ask for help*.”

In contrast to perceived norms indicating community-level stigma against seeking support for mental health issues, most participants expressed personal support for seeing a mental health professional. One participant stated, “You must visit the mental health doctor because he knows about mental health. That’s his job. He will know how to communicate and treat you, and getting treated, you’ll get better”.

### 3.4. Social Interaction May Alleviate Mental Health Symptoms

When asked about helpful methods to address mental health related symptoms, participants identified social connectedness as an important strategy for improving mental health. According to one participant, one should “interact with people in the community. The important thing is avoiding being alone.” Another participant also expressed that, “If sometimes that person goes back to their country that they came from and sees their friends and family, then that person’s sadness stress and anxiety might reduce”.

## 4. Discussion

This study assessed perceptions about mental health symptoms, causes and consequences, and mental health-related beliefs within the community among Kurdish refugees from Iraq in Middle Tennessee. There were three key findings. First, participants identified social isolation and a loss of professional status as major causes of mental illness symptoms. Second, they identified greater social connection as an important element for alleviating symptoms of mental illness. Finally, participants expressed the perception that Kurdish community members do not seek help for mental health issues because they are afraid of gossip and negative perceptions from other members of the community.

The socio-ecological model [26] and the social determinants of health framework [27] support these findings that intersecting social factors influence mental health beliefs, outcomes, and perceived needs. While past research has identified many structural barriers to mental health support, this study builds on past work to situate addressing social connections, social status, and social stigma as possible points of intervention by the local community partner. Refugees experience social isolation when resettling in a new country and a new culture. The challenges of navigating different sociocultural norms add additional psychological stress to refugees’ lives [4]. These ongoing daily stressors of post-resettlement can exacerbate mental health problems for refugees [31]. Findings from this study highlight how refugee-serving organizations could prioritize the importance of social connectedness in mental health and healing through offering group interventions and programs that focus on fostering connections among participants, professional skills training, and relationships with mental health providers who can facilitate discussion around stigma and mental health.

This kind of social intervention to improve mental wellness may also be more palatable to the targeted population than conducting individual mental health interventions. Some limited evidence indicates that family-based mental health interventions [32], mental health interventions that focus on building social capital [33], and group interventions [34] contribute to improved mental health among refugee populations. Participants in this study discussed how others in their own community would think negatively about someone experiencing mental health issues and seeking support. This finding about perceived public stigma among Kurdish refugees adds to the knowledge base about reports of stigma around people who experience or exhibit symptoms of mental health problems within multiple refugee and immigrant populations [12]. Stigma and a lack of understanding about mental health can prevent refugees from seeking mental health services [16]. Moreover, perception of public stigma, or the negative “reaction that the general population has to people with mental illness” [35] (p. 16) can lead to self-stigma, which results in negative attitudes towards mental health services and less willingness to seek treatment [36]. Talking about social connectedness as a method to increase mental wellness may further open up conversation about mental health and reduce mental health-related stigma.

Addressing misperceived social norms about mental health-related stigma and treatment seeking may be another opportunity to reduce stigma and improve mental health in this population. Responses from participants indicated a difference between personal attitudes about mental health issues and perceived attitudes of others about mental health issues. This discrepancy suggests further research is needed to assess the extent to which norms about mental health related stigma and help-seeking are misperceived within the Kurdish refugee community, and whether perceived norms are associated with personal attitudes about mental health issues and others who may need to seek help. Prior research with college students and other young adults in the US has found that perceived public stigma about mental health is associated with personal attitudes, while the prevalence of public stigma towards mental health issues is overestimated [37,38]. Interventions correcting misperceptions about the prevalence of community-level stigma have led to improved attitudes towards help-seeking for mental distress [37,38].

Overall, this study identified social drivers of mental health illness symptoms, needed supports, and barriers to care among Kurdish refugees. However, beliefs about mental health differed to some extent between men and women in this study. Statements by female participants demonstrated greater support for mental illness help-seeking compared to statements by male participants. Prior research on adults in Western countries indicates some differences in help-seeking attitudes based on gender, with women generally exhibiting more positive attitudes towards help-seeking than men [39,40]. Future research should explore those differences, which could help shape how a community research partner and other service providers address mental health with the Kurdish refugee population.

Future research should also explore the levels of mental health literacy within Kurdish refugee populations. Some evidence indicates that Iraqi refugees in Western countries are less likely to be able to identify depression [13] and PTSD [41]. Mental illness identification is an important element of help-seeking [13,41] and could be an additional factor influencing the help-seeking behavior of Kurdish refugees.

This study has limitations. First, study participants represented a small convenience sample, so findings may not generalize to other Kurdish immigrants. The study may also have included individuals who feel more comfortable discussing mental health and illness compared to non-participants. Regardless, this study adds to almost non-existent literature focused on Kurdish refugee mental health. Second, the research study took place during the initial stages of the COVID-19 pandemic. Thus, the context of the pandemic and associated stress, anxiety, and social isolation may have influenced the findings. Finally, interviews were conducted over the phone which may bias results. Interviewers were more limited in their ability to develop rapport with participants and were not able to assess any non-verbal communication. However, the community-engaged research approach helped to mitigate researcher bias by centering the voices and perspectives from members of the Kurdish community throughout the research process, including design, implementation, and analysis.

Despite these limitations, this study addresses gaps in the literature and highlights evidence that will support the development of programs and services targeting gaps around mental health-related care and support for Kurdish refugee populations. Critically, the community-engaged study design prioritized centering the voices of Kurdish refugees from Iraq in describing the causes and consequences of mental health symptoms within their community. In summary, this study allowed for culturally responsive and generative discussion of factors affecting, preventing, and supporting mental health care seeking within a distinct refugee population that has experienced multiple periods of displacement in the past 50 years.

## 5. Conclusions

Refugee populations are at higher risk for mental illness. However, they are less likely to receive mental health services. This qualitative, community-engaged study with Iraqi Kurdish refugees identified a few key themes about the social context of mental health and help-seeking: the loss of social ties and professional qualifications during the resettlement process were perceived to be the main causes of mental illness; fear of gossip and stigma among fellow Kurdish community members prevents help-seeking; and social connection alleviates symptoms of mental illness. These findings offer insight into the sociocultural context of mental health within the Kurdish refugee community and provide guidance in designing mental health interventions around social connectedness for organizations serving the Kurdish refugee community. Assessing the accuracy of perceived norms about stigma and help-seeking, and how it relates to personal help-seeking, could also inform opportunities for interventions to reduce mental health-related barriers within the Kurdish refugee community in the United States.

## Figures and Tables

**Table 1:** GIVE-GET Grid: A Tool for Clarifying Expectations in an Academic–Community Partnership [29].

**Academic Give**	**Academic Get**
Coordination of research study IRB application Data analysis	Deepen community–academic research partnership Opportunity for impactful community research Pilot data to use in procuring additional grant funds
**Community Give**	**Community Get**
Liaison to refugee community Consultation on best practices with community population	Better understanding of mental health stigma and help-seeking behaviors with community Recommendations for enhancing work with community around mental health Deepen community–academic research partnership Pilot data to use in procuring additional grant funds

**Table 2:** Thematic Analysis of Kurdish Refugee Perceptions about Mental Health and Barriers to Help-Seeking.

Theme	Example Participant Quote
Social network loss	“*I am one of those people who left their parents. I lost a lot of my friends. The main reasons [for symptoms of mental illness] are those*.” —male, age 25
	“*When you first come to a different country being far away from your parents, family, and friends will cause a big impact on you*.” —female, age 41
Loss of professional qualifications and social status	“*You spent twenty years getting your college degree and start all over here. Whatever you had in your own country, you will lose it here, for example, your education and your job*.” —female, age 41
	“*Back in Kurdistan you have your degree and language, and you know the lifestyle there…Coming here to everything being different and learning a new language is very hard in the beginning. The environment changes, their degree is worth nothing*.” —female, age 43
Social stigma and fears about community gossip	“*Not going to see counselor, maybe I have done some things wrong, and I do not wish people know about that. Or he might think, people will talk behind his back*.” —male, age 55
	“*We have a Kurdish saying that says it’s okay to walk on an empty stomach but dress sharp. So it’s not important about how you feel, it’s important about how other people look at you. So, this is just a simple example about the eastern society about how they feel and react. So, when they come here, they don’t want to talk about their issues, and they bring their habits here. So, you will try hard to keep your problems away from people. If they find out they might mock and use it against you as a weak point. Therefore, our people, they don’t talk about their mental issues*.” —male, age 58
	“*Maybe that person doesn’t want to visit a mental health professional, so people won’t think that he is mentally ill*.” —male, age 34
	“*The way our society looks at mental issues is way different than Western communities like Europe or the US. It is so crucial to address this with a specialist mental health doctor. You will be confident about him not sharing your thoughts and behaviors with others. He will keep your secret and won’t make fun of you. [With the exception of doctors], even your closest friend will talk behind your back and will blame you for these [symptoms]. Therefore, mentally ill people will not share and will keep it private. [He is afraid that] people in society will blame him and say he is a psychopath and stay far from him. But in European society, [it] is normal to talk and ask for help*.” —female, age 41
Social connectedness to improve mental health	“*[To improve mental health, one should] interact with people in the community. The important thing is avoiding being alone*.” —female, age 23
	“*If sometimes that person goes back to their country that they came from and sees their friends and family, then that person’s sadness, stress, and anxiety might reduce*.” —male, age 25

## Data Availability

A limited dataset is available from the corresponding author upon reasonable request.

## Appendix A

**Instructions to be read to participants prior to beginning interview**

My name is ______ and I will be interviewing you today. We are conducting this interview as part of a research study looking at how people within the Kurdish community respond to stress and anxiety and sadness. The purpose of the interview is to hear from you about what you believe about these issues and what you think individuals should do if they are experiencing stress or anxiety or sadness. The goal is for us to gain a better understanding of what people in the community are experiencing and what the refugee resettlement agency and other refugee-serving organizations can provide to assist people. The interview should last between 30–60 min. We will be making a recording of the interview. The information provided by you, during the interview, will be analyzed, at a later date, by our study team. However, any final report of this information will be done in a group format to ensure we protect individual confidentiality. No individual participants will be identified. Please do not identify yourself in any way during the interview. You do not have to participate in this interview if you do not want to. If you decide to stop, after we begin, that is OK too. If you have any questions, please feel free to ask them now or at any point during the interview.

**Main Questions:**

Before we get into the topic for today’s interview, let’s start with an introductory question. What is your favorite thing about living in the Nashville community? Please remember to not use your or anyone else’s name so as to ensure confidentiality.

We know that some people in the community experience stress, anxiety, and sadness. Work and navigating a new country can be stressful and cause anxiety. Having lost friends or family members or being far away from friends and family members can cause grief and deep sadness. Sometimes the symptoms can be expressed in the body, and sometimes they can manifest in the brain. The next few questions are related to what people in the community think a person should do if they experience any of these symptoms. When we talk about community, we are referring specifically to the Kurdish community.

If a person in your community is experiencing symptoms of stress what do other people in the community say about why the person is experiencing those symptoms?

Probe question: What is the cause (or causes) of this person’s symptoms?

What might make these symptoms change for a person?

What do people in the community say about what the person should do to alleviate the symptoms?

Probe question: What does alleviating the symptoms look like for people in the community?

Repeat for anxiety and sadness sets of symptoms.

Now we will discuss in more detail about who a person might talk to about their sadness/anxiety/stress symptoms, and particularly why a person would or would not speak with a counselor or another person for advice.

Do people in the community talk to someone to relieve their symptoms? Who would that be?

Sometimes, a person who has experienced some of these symptoms will go see a counselor. Why might someone choose to go see a counselor?

What about a mental health professional?

Sometimes, a person who has experienced some of these symptoms would choose to not see a counselor. Why might someone choose not to see a counselor?

What about a mental health professional?

Sometimes, a person who has experienced some of these symptoms might go see a counselor once or twice, but then stop. Why might someone choose to stop seeing a counselor?

What about a mental health professional?

**Wrap up question:** (interviewer to provide a recap of what has been discussed)

If the refugee resettlement agency could offer a service or program that would provide ways for people in the community to alleviate symptoms of stress, anxiety, or sadness, what do you think that should look like?

Is there anything we should have talked about today but didn’t?

Thank you for your willingness to participate in this interview.

List of Symptoms from the Diagnostic and Statistical Manual of Mental Disorders

Please list applicable symptoms for interviewee during questions 2–5.

Stress Symptoms:

Headache

Overeating or undereating

Muscle tension or pain

Restlessness

Chest pain

Lack of motivation or focus

Drug or alcohol misuse

Fatigue

Feeling overwhelmed

Tobacco use

Irritability or anger

Social withdrawal

Stomach upset

Sleep problems

Anxiety Symptoms:

Feeling nervous, restless, or tense

Having a sense of impending danger, panic, or doom

Having an increased heart rate

Breathing rapidly (hyperventilation)

Sweating

Trembling

Feeling weak or tired

Trouble concentrating or thinking about anything other than the present worry

Having trouble sleeping

Experiencing gastrointestinal (GI) problems

Having difficulty controlling worry

Having the urge to avoid things that trigger anxiety

Depression Symptoms:

Feelings of tearfulness, emptiness, or hopelessness

Angry outbursts, irritability or frustration, even over small matters

Loss of interest or pleasure in most or all normal activities

Sleep disturbances, including insomnia or sleeping too much

Tiredness and lack of energy, so even small tasks take extra effort

Reduced appetite and weight loss or increased cravings for food and weight gain

Agitation or restlessness

Slowed thinking, speaking or body movements

Feelings of worthlessness or guilt, fixating on past failures or self-blame

Trouble thinking, concentrating, making decisions, and remembering things

Unexplained physical problems, such as back pain or headaches
